# Advancing the Use of Fecal Sludge for Timelier and Better-Quality Epidemiological Data in Low- and Middle-Income Countries for Pandemic Prevention

**DOI:** 10.1021/acs.est.2c07788

**Published:** 2022-11-01

**Authors:** Petros Chigwechokha, Renée Street, Rochelle H. Holm

**Affiliations:** † Department of Biological Sciences, Malawi University of Science and Technology, P.O. Box 5196, Limbe, Malawi; ‡ Environment & Health Research Unit, South African Medical Research Council, Parow Valley, Cape Town 7505, South Africa; § Environmental Health Department, Faculty of Health Sciences, University of Johannesburg, Johannesburg 2028, South Africa; ∥ Christina Lee Brown Envirome Institute, School of Medicine, University of Louisville, 302 East Muhammad Ali Boulevard, Louisville, Kentucky 40202, United States

**Keywords:** COVID-19, fecal sludge, low- and middle-income countries, pathogen, wastewater-based epidemiology

The availability and accessibility of water, sanitation, and hygiene (WASH) services are key in preventing disease transmission.^1^ Monitoring non-infectious severe acute respiratory syndrome coronavirus 2 (SARS-CoV-2) RNA in wastewater can serve as an early indicator of changes in coronavirus disease (COVID-19) cases in a particular catchment area.^2^ Fecal sludge is excreta held on site (unsewered) in sanitation systems, such as at households, in shared outhouses, and in septic tanks. These on-site sanitation systems, a positive step toward improved household WASH services, are used by 43% of the global population, mainly comprising low- and middle-income countries (LMICs).^1,3^ There is an urgent need to expand decentralized (non-sewered system) surveillance as LMICs are currently being overlooked in pandemic prevention as it is currently focused on SARS-CoV-2 wastewater from centralized (sewered) systems.

Wastewater surveillance of centralized systems presents a vastly distinct set of challenges compared with fecal sludge surveillance. For example, sewers also contain gray water and sometimes stormwater, and wastewater is commonly sampled as a composite volume over a 24 h period, whereas fecal sludge is most commonly grab sampled. Broadening the focus of non-sewered sanitation surveillance (NSSS) should accommodate fecal sludge analysis to support globally relevant public health strategies.

Even before the COVID-19 pandemic, fecal sludge was used as a valuable source of information for understanding community health.^4^ Unfortunately, interest in fecal sludge research has been largely limited to a small group of researchers funded by a few core donors. The pandemic has renewed the focus of both science and policy. Surveillance of pathogens in fecal sludge may need to be adapted and could include *Vibrio cholerae* or poliovirus; however, this should be determined by assessing localized needs. The potential of the public health impact in LMICs can be amplified by using fecal sludge data in addition to clinical data in multipathogen disease surveillance and continuous local monitoring for early warnings of a pandemic.

Research partnerships are required to progress from individual clinical patient samples (which has been happening at a limited scale in healthcare settings) to pooled community samples in terms of fecal sludge. The importance of research partnerships is not new,^5^ but possibly more complex in the Southern African sanitation field with five decades of human waste research.^6^ With no formal global network, international nonprofit institutions can fill this role by bringing together academic research partners and public health officials for interdisciplinary and collaborative research and increased awareness of NSSS. Such partnerships can enable these settings to innovatively adapt academic or healthcare laboratory operations while maintaining high-quality data and also promote peer-reviewed literature originating from resource-limited laboratory settings.

Timelier and better-quality global epidemiological data sets are required for better pandemic preparedness in the future. There is a need in LMICs for the different, but unique, value of NSSS compared to that of wastewater surveillance, built-in capacity at local laboratories that can include fecal sludge as part of continuous multipathogen disease surveillance, and long-term global research collaborations focused on creating systems with data-driven approaches. Challenges will remain, including importation and lengthy delays in supplies and equipment (such as polymerase chain reaction primers and probes that are not manufactured in many LMICs), repurposing existing LMIC laboratory spaces for fecal sludge samples, and limited LMIC access to biosafety level 3 and 4 laboratories for sample processing, thereby limiting pathogen target lists. We recommend that LMICs navigate through these barriers to ensure that fecal sludge surveillance benefits public health, generates local policy-relevant information for future pandemic prevention and control, and holistically supports global WASH services.
